# Specialised Paediatric PAlliativE CaRe: Assessing family, healthcare professionals and health system outcomes in a multi-site context of various care settings: SPhAERA study protocol

**DOI:** 10.1186/s12904-022-01089-x

**Published:** 2022-11-02

**Authors:** Karin Zimmermann, Michael Simon, Katrin Scheinemann, Eva Maria Tinner Oehler, Michèle Widler, Simone Keller, Günther Fink, Stefan Mitterer, Anne-Kathrin Gerber, Stefanie von Felten, Eva Bergstraesser

**Affiliations:** 1 Paediatric Palliative Care and Children’s Research Center CRC, University Children’s Hospital Zurich, Steinwiesstrasse 75, 8032 Zurich, Switzerland; 2 Department Public Health (DPH), Nursing Science, University of Basel, Bernoullistrasse 28, 4056 Basel, Switzerland; 3 Division of Pediatric Oncology – Hematology and Palliative Care, Kinderspital, Kantonsspital Aarau AG, Tellstrasse 25, 5001 Aarau, Switzerland; 4 Department of Health Sciences and Medicine, University of Lucerne, Lucerne, Switzerland; 5 Department of Pediatrics, McMaster Children’s Hospital and University, Hamilton, Canada; 6 Division of Pediatric Heamtology and Oncology, Paediatric Palliative Care, Children’s Hospital, Inselspital, Universitätsspital Bern, Freiburgstrasse 10, CH-3010 Bern, Switzerland; 7 Paediatric Palliative Care, Children’s Hospital Basel, Spitalstrasse 33, 4056 Basel, Switzerland; 8 Paediatric Palliative Care, Children’s Hospital, Inselspital, Universitätsspital Bern, Freiburgstrasse 10, CH-3010 Bern, Switzerland; 9 Department of Epidemiology and Public Health, Swiss Tropical and Public Health Institute, Kreuzstrasse 2, 4123 Allschwil, Switzerland; 10 University of Basel, Basel, Switzerland; 11 Clinical Trial Unit, Department of Clinical Research, University of Basel, Basel, Switzerland; 12 Department of Biostatistics, Epidemiology, Biostatistics and Prevention Institute, University of Zurich, Hirschengraben 84, 8001 Zurich, Switzerland

**Keywords:** Palliative care, Pediatrics, Effectiveness research, Clinical trials, Complex intervention, Study protocol

## Abstract

**Background:**

The number of children and adolescents living with life-limiting conditions and potentially in need for specialised paediatric palliative care (SPPC) is rising. Ideally, a specialised multiprofessional team responds to the complex healthcare needs of children and their families. The questions of, how SPPC is beneficial, for whom, and under what circumstances, remain largely unanswered in the current literature. This study’s overall target is to evaluate the effectiveness of a SPPC programme in Switzerland with respect to its potential to improve patient-, family-, health professional-, and healthcare-related outcomes.

**Methods:**

This comparative effectiveness study applies a quasi-experimental design exploring the effectiveness of SPPC as a complex intervention at one treatment site in comparison with routine care provided in a generalised PPC environment at three comparison sites. As the key goal of palliative care, quality of life - assessed at the level of the patient-, the family- and the healthcare professional - will be the main outcome of this comparative effectiveness research. Other clinical, service, and economic outcomes will include patient symptom severity and distress, parental grief processes, healthcare resource utilisation and costs, direct and indirect health-related expenditure, place of death, and introduction of SPPC. Data will be mainly collected through questionnaire surveys and chart analysis.

**Discussion:**

The need for SPPC has been demonstrated through numerous epidemiological and observational studies. However, in a healthcare environment focused on curative treatment and struggling with limited resources, the lack of evidence contributes to a lack of acceptance and financing of SPPC which is a major barrier against its sustainability. This study will contribute to current knowledge by reporting individual and child level outcomes at the family level and by collecting detailed contextual information on healthcare provision. We hope that the results of this study can help guiding the expansion and sustainability of SPPC and improve the quality of care for children with life-limiting conditions and their families internationally.

**Trial registration:**

Registered prospectively on ClinicalTrials.gov on January 22, 2020. NCT04236180

**Protocol version:**

Amendment 2, March 01, 2021.

## Background

Following ongoing medical advances and improved diagnosis and coding, the number of children and adolescents living with life-limiting conditions is rising drastically [1]. Estimates from the United Kingdom showed a prevalence of 26.7 per 10,000 children aged 0–19 years in 2000/2001, and a prevalence of 66.4 per 10,000 in 2017/2018 [1]. Many of these children and adolescents need palliative care (PC) services. A new consensus describes PC as “the active holistic care of individuals across all ages with serious health related suffering because of severe illness and especially of those near the end of life. It aims to improve the quality of life of patients, their families, and their caregivers.” ([2], p. 761). As a special and highly complex subfield of PC, paediatric PC (PPC) is concerned with the support and involvement of the entire family, and aims to impact the patient-, family and health system levels. Ideally, to meet the complex healthcare needs of children and their families, a specialised multiprofessional team will be available, i.e. PPC services are offered by healthcare professionals specifically trained and working in PPC in the context of a dedicated programme setting [3].

Specialised PPC (SPPC) programmes most commonly offer a consultative model of care, i.e. by a specialised multiprofessional PPC team that either provides direct patient care and family support that goes beyond the affected child’s eventual death or provides support and advice to the child’s primary care team in and outside of the hospital [4]. In such a setup, medical specialists (mainly physicians), e.g. neurologists or oncologists refer their patients and families in need. Referrals are mainly driven by symptom burden and the burden of the child’s condition on the entire family. However, the referral practices also depend on personal attitudes and motivation of the referrers from the medical specialties towards SPPC. Furthermore, the referrer’s perception of evidence supporting SPPC can be considered as a contextual factor influencing referral practices [5] and it is well recognised that compelling scientific evidence on the effectiveness of SPPC is scarce [6–8].

### State of research

The question of whether PC in general is associated with improved patient and caregiver outcomes has been studied and summarised in a meta-analysis of 43 randomised controlled trials with data on 12′731 adult patients [9]. PC interventions were associated with improvements in patient quality of life (QOL) and symptom burden, however, results regarding caregiver outcomes were inconsistent [9]. In PPC, patient-reported outcome assessments are not normally feasible due to the patient’s age or cognitive state. Therefore, in paediatric research, proxy reports from parents are commonly used [10]. A systematic review including eight observational studies found improved QoL in children and family members, improved symptom control, and a positive impact on place of care and family support [11]. The burdens on family members are substantial. Investigating the impact of chronic health conditions on siblings psychological functioning and well-being, Vermaes et al. noted, that the siblings of children with life-threatening conditions appeared especially prone to psychological problems [12]. A recent scoping review, including 34 studies concluded that the experiences these siblings make, impact the way they cope with stress [13]. We found no studies about the influence of SPPC services on the QOL of these siblings.

For healthcare professionals (HCPs), providing compassionate care for children with life-limiting conditions and their families is emotionally demanding. High expectations, lack of support and a sense of inadequate preparation, representing demands from the health care system, can lead to stress in care personnel [14]. One purpose of a consultative SPPC service model is to provide support and advice to each child’s/family’s primary care team, which might ease the care burden and positively influence the QOL of healthcare professionals not specialised in PPC. In a US study of 314 diverse HCPs, 39% were gaged at risk for compassion fatigue (CF), a construct within professional QOL linked to impaired quality of care provision [14, 15].

More evidence related to improved process outcomes at the service level and healthcare resource utilisation is available regarding children/families receiving SPPC. A recent systematic review including 14 cohort studies and one case series found, that the receipt of PPC was associated with a decrease in intensive care use and high-intensity end-of-life care. Regarding hospital admissions, length of stay in hospital, resuscitation orders, and the proportion of hospital and home deaths results were less conclusive [16]. Evidence on healthcare resource utilisation and costs among children, who had accessed a PPC programme versus those, who had not, was summarised in a systematic review [17]. Children enrolled in PPC had fewer hospital admissions, with most studies also showing shorter hospital stays. Conflicting results arose regarding the proportion of patients who received critical care, and calculations of overall healthcare costs were inconclusive [17]. Lower medical costs among PPC recipients through the reduction of healthcare utilisation, however, were recorded in more recent studies [18, 19].

The effectiveness of SPPC as a complex intervention is potentially influenced by many accompanying factors through mechanisms which are not well understood. Important potential mediators of the receipt of SPPC, e.g., the family’s adaptability, should be considered. Based on McCubbin & McCubbin’s resilience model of family stress, adjustment and adaptation, a positive relationship has been described between the family’s adaptation and the family system, i.e., coherence and family hardiness [20]. Associated with adaptation and considered as central to successful coping with family stressors is the construct of sense of coherence. This refers to an orientation between family members, that makes their reactions to internal and external stimuli structured and predictable, providing resistance resources for handling stimuli, and fostering a sense that life’s challenges are meaningful [21]. Family hardiness has been described as a family resource and acknowledged as a mediator in the relationship between stress and illness. As such it has also been related to family members’ health and QOL [22].

### Rationale

As more and more countries have recognised the need for SPPC at the policy level, the international development and implementation of PPC programmes have skyrocketed. However, many of those programmes struggle with the transition into routine in- and outside hospital care and therefore sustainability [4]. The question, of how SPPC is beneficial for whom and under what circumstances, remains largely unanswered as validation of innovative care programmes in controlled studies is lacking [18]. Determining clinical effectiveness will require prospective studies using comparison groups to establish the relationship between the receipt of SPPC and selected outcomes on the client and service levels [23]. Additionally, the question of when to initiate SPPC, and the outcomes of early vs. late introduction of SPPC have never been compared, a comparison of the two has been judged a priority in PPC research [24]. Reporting on outcome metrics at the family level and addressing contextual information on healthcare provision has the potential to guide the expansion and sustainability of services and improve the quality of care for children with life-limiting conditions and their families internationally.

### Objectives

The SPhAERA study’s overall target is to evaluate the effectiveness of SPPC and to report on its potential to improve patient-, family-, health professional-, and healthcare-related outcomes. Specifically,

- to explore how SPPC influences the QOL of patients (including their symptom severity and distress), parents and siblings (including grief processes);
- to explore how the availability of an SPPC team influences the QOL of healthcare professionals not specialised in PPC;
- to determine whether the provision of SPPC reduces the utilisation of healthcare resources and costs;
- to determine whether the provision of SPPC reduces direct and indirect health-related costs for families; and
- to evaluate the introduction of SPPC and validation of the Paediatric Palliative Screening Scale (PaPaS-Scale) [25].

## Methods

### Design and setting

**Fig. 1:**
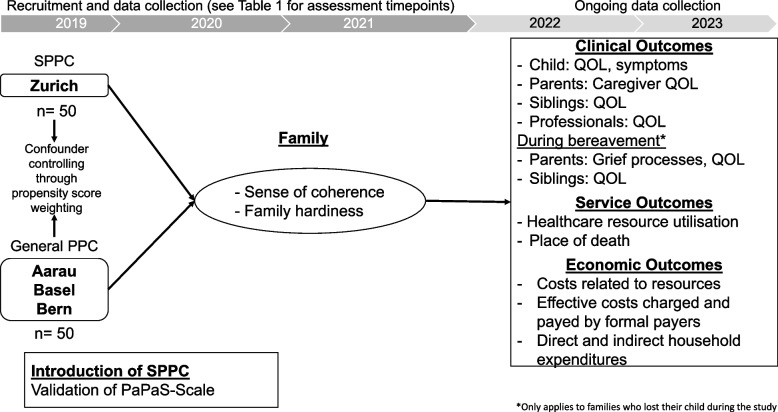
Overview of the study’s setup, timeline and outcomes

This study applies a quasi-experimental design within the framework of comparative effectiveness research, exploring the effectiveness of SPPC as a complex intervention in comparison with routine care provided in a non-specialised PPC environment. The study takes place at four study sites: the largest Swiss University Children’s Hospital with a longstanding established dedicated SPPC programme serves as the intervention site, two other Swiss university children’s hospitals and a Cantonal children’s hospital providing general PPC and are just developing SPPC comprise the comparison sites. Recruitment took place between November 2019 and May 2022, and the longitudinal data collection is ongoing until May 2023. An overview of the study’s setup, timeline and outcomes is provided in Fig. 1.

### Participants and recruitment

#### Patients and families

Children suffering from a life-limiting condition and potentially in need of SPPC, their parents and siblings, as applicable, were eligible to enter the study under the following inclusion criteria: 1) children, aged 0–18 years; parents (mothers and/or fathers) of included children; 2) siblings, aged 8–18 years, of included families; 3) proficiency in German or French language. Neonates with medical complications due to prematurity and/or birth complications and treated in a neonatal intensive care unit and patients enrolled in the SPPC programme with an expected life expectancy of < 48 h were excluded due to limited exposure time. Additionally, children and their families under child protection regulations were not eligible.

For the intervention site, all children entering the SPPC programme, after referral by a member of the frontline care team (usually a physician), were prospectively and consecutively screened for eligibility and invited for study participation. Recruitment was then performed by a member of the SPPC team within the first 2 weeks of SPPC initiation.

For the comparison sites, recruitment started in February 2020 and was performed by specialists of various medical disciplines, e.g. neurology or oncology after evaluating the potential need for SPPC of their patients. This need was defined per indication criteria of the SPPC programme at the intervention site and read as follows: 1) increase in (unplanned) hospital admissions during the last months; 2) adverse medical events from which the child is not recovering completely; 3) increasing symptom burden; unsatisfactory response to treatments; 4) conflicting treatment goals; 5) estimated life expectancy less than 6–12 months; 6) patient’s/parents’ wish for PC support [26].

#### Healthcare professionals (HCPs)

All HCPs of the following professions working at the study sites or in collaboration with them and involved in the care of the population under study were invited for study participation: physicians, registered nurses, health care assistants, psychologists, social workers, physical therapists, occupational therapists, nutritionists, pastoral workers, logo therapists, social pedagogues, remedial teachers and certified social care workers. To ensure their involvement in PPC, the following inclusion criteria were defined: 1) having cared for patients and their families in the PC phase during a minimum of ten shifts (day/late-shift) or ten consultations and/or 2) having cared for a minimum of two patients in the end-of-life phase and their families or after the death of the child; 3) employment in their institution for a minimum of 3 months. Members of the SPPC team at the intervention site were excluded due to their specialisation and dedicated SPPC activities including intervention provision.

Recruitment took place in two cycles: first cycle beginning of 2021 and second cycle beginning of 2022. All HCPs were invited via the written study information which they received through a local coordinator at their institution. The HCPs were instructed to autonomously check their eligibility for study participation based on the criteria listed above.

### Intervention and comparison

The service of a SPPC team is considered as a complex intervention with components on various levels and of variable dosing, based on the needs of each individual patient and her/his family. All services provided to patients/families by a member of the multiprofessional SPPC team at the University Children’s Hospital in Zurich are considered as study intervention. This includes direct medical, nursing, social, and psychological and spiritual consultations of the patient/family, as well as patient−/family-related consultation of the frontline care team. Bereavement support, as integrated part of SPPC is routinely offered at the individual or group level as appropriate for parents and siblings. The programme at the intervention site offers full 24/7 services from the SPPC team’s physicians, nurses and psychologists and includes home visits.

For patients/families in the comparison group (Aarau, Basel, Berne) routine care is provided as per established paediatric standards in a generalised PPC environment (provided by disease specific specialists with some PPC training [3]) already in place at all study sites. In all of the three comparison sites a PPC team is available since 2019/2020. However, these developing teams differ on the level of capacity, i.e. few human resources and mostly professionals with basic training and experience in PPC without being fully engaged in PPC, no 24/7 coverage, and limited psychologist and bereavement support.

Assignment to study groups will happen naturally, determined through the recruiting study sites. As this study is conducted in a natural environment with continuous development of care practices, adaptations of care structures and processes at all study sites are possible and probable during the duration of the study. No restraints of this natural evolution will be superimposed by the study. Care context is assessed on yearly basis in all four participating study sites, described and used as instrumental variables estimation as applicable.

### Outcomes and procedures

Improving and maintaining QOL is the core intention of PC and is the main outcome of this comparative effectiveness study. For an overview of the study’s setup and timeline, outcomes and measurements, and assessment timepoints see Fig. 1 and Tables 1 and 2.

**Table 1:** Overview of outcomes, measurements and data collection time points

	Data source / Instrument	Domains / Dimensions	Data Collection
**Clinical Outcomes:**			
**Primary outcome:**			
Caregiver QOL	Quality of Life in Life Threatening Illness–Family Carer Version (**QOLLTI-F**): 16 items [27]	State of carer, patient wellbeing, quality of care, outlook, environment, finances, and relationships	Baseline at study entry and dynamically after that. Self-report. Mothers, fathers
**Secondary outcomes during the child’s PC phase:**			
Child’s QOL	DISABKIDS Chronic Generic Measure - **DCGM-37**: 37 items [28]	Mental, social, and physical, based on the WHO conceptualisation of health-related QOL.	Baseline at study entry and dynamically after that. Self-report (7 years and older) or proxy report (parent).
Child’s symptoms	Memorial Symptom Assessment Scale (**MSAS**): 30 or 8 items (depending on child’s age) [29, 30]	Prevalence, severity, distress	
Siblings QOL	KIDSSCREEN-27: 27 items [31]	Physical well-being, psychological well-being, autonomy & parent relation, peers & social support, school environment	Baseline at study entry and dynamically after that. Self-report (8 years and older)
Professional’s QOL	Professional QOL (**ProQOL**): 30 items [32]	Compassion satisfaction, compassion fatigue, i.e. burnout and work-related secondary trauma	Cross-sectional. Self-report. HCPs not specialised in PPC but **involved in direct patient care of PPC patients**.
**Secondary outcomes during bereavement:**			
Grief processes	Würzburger Trauerinventar” (**WüTi**): 24 items [33]	Acute emotional and cognitive impairments, general personal development/growth, feelings of guilt and self-reproaches, increase of sensitivity/empathy for others, closeness to the late person	1 month after the child’s death and three-monthly after that. Self-report. Mothers, fathers
Parental QOL	The WHO Quality of Life (**WHOQOL-BREF**): 26 items [34]	Physical, psychological, social relationships, environment	
Siblings QOL	KIDSSCREEN-27: 27 items [31]	Physical well-being, psychological well-being, autonomy & parent relation, peers & social support, school environment	1 month after the sibling’s death and three-monthly after that. Self-report (8 years and older).
**Mediators of SPPC Outcomes:**			
Sense of coherence (SOC)	Family Sense of Coherence (**FSOC**): 26 items [21]	Comprehensibility, manageability, meaningfulness	Cross-sectional at study enrolment. Structured interview. Mothers, fathers
Family hardiness (FH)	Family Hardiness Index (**FHI**): 20 items [22]	Commitment (internal strengths and cooperativeness), challenge (resourcefulness and willingness to learn), control (sense of having control over life circumstances)	Cross-sectional at study enrolment. Structured interview. Mothers, fathers
**Service Outcomes:**			
Healthcare resource utilisation	Routine data	Number of hospital admissions including number of emergency and/or outpatient consultations and number of admissions to a paediatric intensive care unit (PICU), number of resuscitations, number of invasive procedures e.g. surgery and imaging requiring sedation, total length of hospital stay (LOS) per admission, number of days receiving professional community home care services	Chart review, continuously during the child’s PC phase.
Place of death	Routine data	PICU, hospital ward, home, and other	Chart review at time of death
**Economic outcomes:**			
Economic analysis	Routine data Household data	Costs related to healthcare resources utilised Effective costs charged and paid by formal payers Direct and indirect health-related household expenditures: Out of pocket payments, changes in employment status and income, financial support	Cost data retrieved from hospitals and formal payers for the time of study participation. Baseline at study entry and dynamically after that. Self-report. Mothers, fathers
**Introduction of SPPC:**			
Introduction of SPPC and Validation of the PaPaS-Scale	Paediatric Palliative Screening Scale (**PaPaS-Scale**): 11 items [25]	Trajectory of disease and impact on daily activities of the child, expected outcome of treatment directed at the disease and burden of treatment, symptom and problem burden, preferences/needs of patient or parents / preferences of HCPs, estimated life expectancy	Cross-sectional for each family and child older than 1 year of age at study entry.
**HCPs Outcomes**			
Professional Quality of Life	ProQOL Version 5: 30 items [35]	Compassion fatigue, i.e. burnout and secondary traumatic stress, and compassion satisfaction	Cross-sectional. Self-report. HCPs not specialised in PPC and involved in direct care of PPC or end-of-life patients.

**Table 2:** Study visits and assessments

Study Periods	Care Timepoint									Bereavement Timepoint				
Timepoint^**a**^	Screening/ Baseline	CT1	CT2	CT3	CT4	CT5	CT6	CT7	CT8	Death of child	BT1	BT2	BT3	BT4
Time interval in days (Reference = 0)	0	15	30	60	90	120	150	240	330	0	30	120	210	300
Informed Consent / Demographic Data	**x**													
Inclusion / Exclusion Criteria	**x**													
Introduction of SPPC (PaPaS-Scale)	**x**													
Sense of coherence (FSOC)	**x**													
Family hardiness (FHI)	**x**													
Caregiver QOL (QOLLTI-F)	**x**	**x**	**x**	**x**	**x**	**x**	**x**	**x**	**x**					
Child’s QOL (disabkids)	**x**		**x**	**x**	**x**	**x**	**x**	**x**	**x**					
Child’s symptoms (MSAS)	**x**	**x**	**x**	**x**	**x**	**x**	**x**	**x**	**x**					
Siblings QOL (KIDSSCREEN-27)	**x**	**x**	**x**	**x**	**x**	**x**	**x**	**x**	**x**					
Healthcare resource utilisation and costs		**x**	**x**	**x**	**x**	**x**	**x**	**x**	**x**	**x**				
Direct and indirect health-related expenditure	**x**		**x**	**x**	**x**	**x**	**x**	**x**	**x**			**x**		**x**
Professional’s QOL (ProQOL)									**x**^**b**^					
Place of death										**x**				
Grief processes (WüTi)											**x**	**x**	**x**	**x**
Parental QOL (WHOQOL-BREF)											**x**	**x**	**x**	**x**
Siblings QOL (KIDSSCREEN-27)											**x**	**x**	**x**	**x**

^a^The timeframe to complete the questionnaire is 1 week

^b^Professional’s QOL will be assessed after the first year of recruitment and after the second year, i.e. end of recruitment phase

#### Patients and families

Since a large proportion of the study population is young or cognitively impaired, we defined caregiver QOL as the primary outcome. Each participating patient/family is followed for a maximum time of 1 year as long as the child is alive. Assessment timepoints are dynamic, starting with two bi-weekly assessments, followed by four monthly assessments and three-monthly after that. For families, who lose their child during the study, bereavement follow-up continues for another year with four assessments. All outcomes on the family level are assessed as surveys with self-reported and validated questionnaires on paper. Questionnaires are distributed in the hospital or sent home by the study team in case the child is at home at the time of a study assessment. Outcomes on the patient level are assessed as proxy measures through the parents, unless the patient is capable to report her−/himself. Siblings’ QOL is only assessed, if they are able to self-report.

Service outcomes are assessed through the collection of routine data via chart review at assessment timepoints. This includes patient-specific information related to healthcare resource utilization, e.g. hospital admissions and length of stays, procedures, and diagnostic information and date of death. Economic outcomes are assessed through queries for each patient for costs on the healthcare system level (hospitals, formal payers) and through self-reported direct and indirect health-related expenditure data (questionnaire) from participating families.

#### HCPs

HCPs’ professional QOL was retrospectively assessed for 1 year back after the first and second study year. All HCPs who returned their informed consent received the questionnaire as a hard copy through institutional or postal mail.

All study participants are withdrawn from the study in the case of consent withdrawal or relevant non-adherence to study procedures, i.e. failure to complete questionnaires. No specific follow-up beyond the date of withdrawal/discontinuation is performed and no more data is collected. All data collected up to study withdrawal/discontinuation will be considered for analysis.

### Sample size and statistical analyses

We hypothesise that SPPC positively influences the QOL of the caring parents. The null hypothesis is that QOL does not differ between parents of patients in the SPPC programme and the comparison group. The sample size (number of paediatric patients) was calculated to be able to show a difference of 1 point in the QOLLTI-F score [27] 1 month after study inclusion. The calculations are based on an expected mean QOLLTI-F of 5.5 (on a scale from 0 to 10) and an expected standard deviation of the QOLLTI-F score of 1.7, as reported in Cohen et al. [27]. Note that similar values of mean ± SD, i.e., 5.8 ± 1, were observed by Groh et al. [36].

Sample size was calculated using a resampling method. Each sample size, n i = 1,...,41 = 10, ..., 250, was evaluated by simulating *R* = 499 times the QOLLTI-F of n_i_ individual pairs of care giving parents from a multivariate normal distribution, using a correlation of 0.8 between parents of the same patient. For each sample size n_i_, half of the patients were allocated to the SPPC group and comparison group, with mean QOLLTI-F of 6 and 5, respectively, and a (within group) standard deviation of 1.7. Assuming that only one parent participates in the study for 30% of the patients, this proportion of simulated QOLLTI-F scores for the second parent was omitted, resulting in simulated QOLLTI-F scores for a pair of parents for 70% of the patients, and for only one parent for the remaining 30% of patients. The difference in QOLLTI-F between parents of SPPC vs. comparison patients was then estimated with a linear mixed-effects model with group (SPPC vs. comparison) as fixed factor, modelling a random intercept per patient (to account for the non-independence of parents from the same patient). Sample size was set to ensure at least a power, 1 – β, of 0.8 at a significance level, α, of 0.05.

For this study, a total of 98 paediatric patients should be recruited (i.e., 49 from the SPPC programme and 49 for comparison) to ensure a total of 98 evaluable pairs of care giving parents, considering a drop-out rate (i.e., proportion of patients who died or withdrew informed consent before QOLLTI-F could be assessed at least once) of 10%. Sample size was estimated using R (Version 3.5.0) [37], using the R packages nlme [38] and sse [39].

#### Primary and secondary analyses

The primary outcome QOLLTI-F of the care-giving parents 1 month after study inclusion will be analysed by a linear mixed-effects model with QOLLTI-F at baseline and group (SPPC vs. comparison) as fixed effects. A random intercept will be modelled per patient (to account for the non-independence of parents from the same patient). In addition, all measurements of QOLLTI-F taken at different follow-up visits will be analysed by a linear mixed-effects model as above, but with an additional random intercept per parent (to additionally account for the non-independence of multiple measurements per parent).

To adjust the effect size estimate for SPPC vs. comparison for potential confounders, we will use propensity score weighting. Potential confounders were already identified and include characteristics of the patient (diagnosis, age, sex), disease duration, family system (sense of coherence, family hardiness), sociodemographic variables of the family (e.g. parental living situation, family income), and contextual information (e.g. study site characteristics). As a sensitivity analysis, we will add the most important confounders as covariates in the statistical models (analysis of covariance approach).

Secondary endpoints will be analysed by linear mixed-effects models or generalised linear mixed-effects models (in case of non-normal error distribution). Normal linear models or generalised linear models may be used for secondary outcomes measured only once per patient. Sensitivity analyses will be performed as applicable and appropriate.

Missing measurements of the primary outcome, QOLLTI-F at 1 month, will be multiply imputed, based on measurements taken at 2 weeks (first follow-up measurement) and available patient characteristics.

### Data management, monitoring and risks

The sponsor-investigator is implementing and maintaining quality assurance and quality control systems to ensure that the study is conducted and data are generated, documented (record), and reported in compliance with the protocol, good clinical practice, and applicable regulatory requirement(s). Data extracted from the patient charts will be entered into an internet-based secure data base secuTrial® developed in agreement to the Good Clinical Practice guidelines provided by the Clinical Trials Centre Zurich. Person data will be pseudonomised through secuTrial®. Local coordinators will be assigned to assist with and facilitate logistics concerning the availability of medical charts and workspace in each participating study site. Data collectors will receive instructions and training before the start of data collection to assure and enhance data quality. Furthermore about 5% of the charts will be randomly chosen and data will be collected by two different people for quality checking.

A quality assurance audit/inspection of this study may be conducted by the competent ethics committee. The quality assurance auditor/inspector will have access to all medical records, the investigator’s study related files and correspondence, and the informed consent documentation that is relevant to this clinical study. We consider the risk for participants in this study as minimal. Allocation to intervention and comparison groups is determined by the natural environment of the four study sites’ care services.

## Discussion

This study evaluates the effectiveness of SPPC and its impact on patient-, family-, health professional-, and healthcare-related outcomes. The study will contribute to current knowledge by providing relevant outcome data based on the assessment of SPPC services within a comparative effectiveness research framework. Reporting on outcome metrics at the family level and providing detailed contextual information on healthcare provision has the potential to guide the expansion and sustainability of services and improve the quality of care for children with life-limiting conditions and their families internationally.

Determining clinical effectiveness will require prospective studies using comparison groups to establish the relationship between the receipt of SPPC and selected outcomes on the client and service levels [23]. However, the classical randomised controlled trial study design with its origin in establishing the efficacy of new drugs under controlled situations falls short in evaluating the effectiveness of a complex intervention in a real-world setting. Effectiveness research broadens the design options for evaluating interventions by possibly loosening up some of the rigorous control measures mandatory in a randomised controlled trial and therefore trading off strong internal validity in favour of generalisability (external validity) [40]. The consequence of less rigorous controls is an increased risk of confounding, which may limit causal interpretation of the study results.

Dealing with potential imbalance between intervention and comparison group will likely be this study’s greatest challenge. We will take all possible and reasonable measures, e.g. propensity scoring, multilevel modelling to ensure justified group comparisons and interpretation. The study’s rich longitudinal data over a period of approximately 1 year will allow a unique reporting on courses of illness of the child, of the QOL of close family members and the family’s financial hardships.

A variety of outcome indicators were used to assess the impact of SPPC so far. Widger et al. found 82 different indicators reported through 46 studies. Among this large number, indicators such as location of death, length of stay in hospital and number of hospital admissions, only 22 indicators were reported in at least two studies [41]. Many of these so far used indicators are also assessed in our study. Others, such as our main outcome of QOL on different levels are less investigated, limiting comparability with our study results.

The main results of this study will be communicated to patients, their family, and the involved healthcare professionals by a letter in lay language. The study group will make every endeavour to publish the data in peer-reviewed journals and we are convinced that this study’s comparative effectiveness and longitudinal approach will generate new meaningful evidence to advance the field of PPC internationally.

## Data Availability

Not applicable.

## Abbreviations

DCGM: DISABKIDS Chronic Generic Measure
FHI: Family Hardiness Index
FSOC: Family Sense of Coherence
HCP: Health Care Professional
MSAS: Memorial Symptom Assessment Scale
QOL: Quality of Life
QOLLTI-F: Quality of Life in Life Threatening Illness–Family Carer
PaPaS-Scale: Paediatric Palliative Screening Scale
PC: Palliative Care
PPC: Paediatric Palliative Care
ProQOL: Professional QOL
SPPC: Specialised Paediatric Palliative Care
WHOQOL: The WHO Quality of Life
WüTi: Würzburger Trauerinventar

## References

[1] Fraser LK, Gibson-Smith D, Jarvis S, Norman P, Parslow RC (2021). Estimating the current and future prevalence of life-limiting conditions in children in England. Palliat Med.

[2] Radbruch L, De Lima L, Knaul F, Wenk R, Ali Z, Bhatnaghar S, Blanchard C, Bruera E, Buitrago R, Burla C (2020). Redefining palliative care-a new consensus-based definition. J Pain Symptom Manag.

[3] Benini F, Papadatou D, Bernada M, Craig F, De Zen L, Downing J, Drake R, Friedrichsdorf S, Garros D, Giacomelli L (2022). International standards for pediatric palliative care: from IMPaCCT to GO-PPaCS. J Pain Symptom Manag.

[4] Feudtner C, Womer J, Augustin R, Remke S, Wolfe J, Friebert S, Weissman D (2013). Pediatric palliative care programs in children's hospitals: a cross-sectional national survey. Pediatrics.

[5] Damschroder LJ, Aron DC, Keith RE, Kirsh SR, Alexander JA, Lowery JC (2009). Fostering implementation of health services research findings into practice: a consolidated framework for advancing implementation science. Implement Sci.

[6] Downing J, Knapp C, Muckaden MA, Fowler-Kerry S, Marston J, Committee IS (2015). Priorities for global research into children's palliative care: results of an international Delphi study. BMC Palliat Care.

[7] Thienprayoon R, Jones E, Humphrey L, Ragsdale L, Williams C, Klick JC (2022). The pediatric palliative improvement network: a national healthcare learning collaborative. J Pain Symptom Manag.

[8] Feudtner C, Rosenberg AR, Boss RD, Wiener L, Lyon ME, Hinds PS, Bluebond-Langner M, Wolfe J (2019). Challenges and priorities for pediatric palliative care research in the U.S. and similar practice settings: report from a pediatric palliative care research network workshop. J Pain Symptom Manag.

[9] Kavalieratos D, Corbelli J, Zhang D, Dionne-Odom JN, Ernecoff NC, Hanmer J, Hoydich ZP, Ikejiani DZ, Klein-Fedyshin M, Zimmermann C (2016). Association between palliative care and patient and caregiver outcomes: a systematic review and meta-analysis. JAMA.

[10] Huang IC, Shenkman EA, Madden VL, Vadaparampil S, Quinn G, Knapp CA (2010). Measuring quality of life in pediatric palliative care: challenges and potential solutions. Palliat Med.

[11] Mitchell S, Morris A, Bennett K, Sajid L, Dale J (2017). Specialist paediatric palliative care services: what are the benefits?. Arch Dis Child.

[12] Vermaes IP, van Susante AM, van Bakel HJ (2012). Psychological functioning of siblings in families of children with chronic health conditions: a meta-analysis. J Pediatr Psychol.

[13] Tay J, Widger K, Stremler R. Self-reported experiences of siblings of children with life-threatening conditions: a scoping review. J Child Health Care. 2021:13674935211026113. 10.1177/13674935211026113. 10.1177/13674935211026113 PMC9667075 34116616

[14] Sinclair S, Raffin-Bouchal S, Venturato L, Mijovic-Kondejewski J, Smith-MacDonald L (2017). Compassion fatigue: a meta-narrative review of the healthcare literature. Int J Nurs Stud.

[15] Robins PM, Meltzer L, Zelikovsky N (2009). The experience of secondary traumatic stress upon care providers working within a children's hospital. J Pediatr Nurs.

[16] Lin SC, Huang MC, Yasmara D, Wuu HL (2021). Impact of palliative care on end-of-life care and place of death in children, adolescents, and young adults with life-limiting conditions: a systematic review. Palliat Support Care.

[17] Conte T, Mitton C, Trenaman LM, Chavoshi N, Siden H (2015). Effect of pediatric palliative care programs on health care resource utilization and costs among children with life-threatening conditions: a systematic review of comparative studies. CMAJ Open.

[18] Chong PH, De Castro Molina JA, Teo K, Tan WS (2018). Paediatric palliative care improves patient outcomes and reduces healthcare costs: evaluation of a home-based program. BMC Palliat Care.

[19] Lysecki DL, Gupta S, Rapoport A, Rhodes E, Spruin S, Vadeboncoeur C, Widger K, Tanuseputro P (2022). Children’s health care utilization and cost in the last year of life: a cohort comparison with and without regional specialist pediatric palliative care. J Palliat Med.

[20] McCubbin MA, McCubbin HI, Danielson CB, Hamel-Bissel B, Winstead-Fry P (1989). Families coping with illness: the resiliency model of family stress, adjustment and adaptation. Families, health & illness: perspectives on coping and intervention.

[21] Antonovsky A, Sourani T (1988). Family sense of coherence and family adaptation. J Marriage Fam.

[22] Persson C, Benzein E, Arestedt K (2016). Assessing family resources: validation of the Swedish version of the family hardiness index. Scand J Caring Sci.

[23] Osenga K, Postier A, Dreyfus J, Foster L, Teeple W, Friedrichsdorf SJ (2016). A comparison of circumstances at the end of life in a hospital setting for children with palliative care involvement versus those without. J Pain Symptom Manag.

[24] Baker JN, Levine DR, Hinds PS, Weaver MS, Cunningham MJ, Johnson L, Anghelescu D, Mandrell B, Gibson DV, Jones B (2015). Research priorities in pediatric palliative care. J Pediatr.

[25] Bergstraesser E, Hain RD, Pereira JL (2013). The development of an instrument that can identify children with palliative care needs: the Paediatric Palliative Screening Scale (PaPaS Scale): a qualitative study approach. BMC Palliat Care.

[26] Zurich UCsH. Konzept Pädiatrische Palliative Care. Zurich: University Children's Hospital Zurich; 2016. p. 22.

[27] Cohen R, Leis AM, Kuhl D, Charbonneau C, Ritvo P, Ashbury FD (2006). QOLLTI-F: measuring family carer quality of life. Palliat Med.

[28] Baars RM, Atherton CI, Koopman HM, Bullinger M, Power M, group D. The European DISABKIDS project: development of seven condition-specific modules to measure health related quality of life in children and adolescents. Health Qual Life Outcomes. 2005;3:70. 10.1186/1477-7525-3-70 PMC1326227 16283947

[29] Collins JJ, Byrnes ME, Dunkel IJ, Lapin J, Nadel T, Thaler HT, Polyak T, Rapkin B, Portenoy RK (2000). The measurement of symptoms in children with cancer. J Pain Symptom Manag.

[30] Collins JJ, Devine TD, Dick GS, Johnson EA, Kilham HA, Pinkerton CR, Stevens MM, Thaler HT, Portenoy RK (2002). The measurement of symptoms in young children with cancer: the validation of the memorial symptom assessment scale in children aged 7-12. J Pain Symptom Manag.

[31] KIDSCREEN - health related quality of life questionnaire for children and young people and their parents. 2011. https://www.kidscreen.org/. Accessed 30 May 2017.

[32] ProQOL, Professional Quality of Life. 2021. http://www.proqol.org. Accessed 22 Sept 2022.

[33] Wittkowski J (2013). Würzburger Trauerinventar: Mehrdimensionale Erfassung des Verlusterlebens.

[34] Angermeyer MC, Kilian R, Matschinger H (2000). WHOQOL-100 und WHOQOL-BREF: Handbuch für die deutschsprachige Version der WHO Instrumente zur Erfassung von Lebensqualität.

[35] Stamm BH. The concise ProQOL manual. 2nd ed. Pocatello: https://proqol.org/proqol-manual; 2010.

[36] Groh G, Borasio GD, Nickolay C, Bender HU, von Luttichau I, Fuhrer M (2013). Specialized pediatric palliative home care: a prospective evaluation. J Palliat Med.

[37] R Core Team (2018). R: a language and environment for statistical computing.

[38] Pinheiro J, Bates D, Team RC (2018). nlme: linear and nonlinear mixed effects models. R package version 3.1–137 edn.

[39] Fabbro T (2018). sse: sample size estimation. R package version 0.6–4 edn.

[40] Portney LG (2020). Foundations of clinical research: applications to evidence-based practice.

[41] Widger K, Medeiros C, Trenholm M, Zuniga-Villanueva G, Streuli JC (2019). Indicators used to assess the impact of specialized pediatric palliative care: a scoping review. J Palliat Med.

