# Correction: Specialised Paediatric PAlliativE CaRe: Assessing family, healthcare professionals and health system outcomes in a multi-site context of various care settings: SPhAERA study protocol

**DOI:** 10.1186/s12904-023-01136-1

**Published:** 2023-02-22

**Authors:** Karin Zimmermann, Michael Simon, Katrin Scheinemann, Eva Maria Tinner Oehler, Michèle Widler, Simone Keller, Günther Fink, Stefan Mitterer, Anne-Kathrin Gerber, Stefanie von Felten, Eva Bergstraesser

**Affiliations:** 1 Paediatric Palliative Care and Children’s Research Center CRC, University Children’s Hospital Zurich, Steinwiesstrasse 75, 8032 Zurich, Switzerland; 2 Department Public Health (DPH), Nursing Science, University of Basel, Bernoullistrasse 28, 4056 Basel, Switzerland; 3 Division of Pediatric Oncology – Hematology and Palliative Care, Kinderspital, Kantonsspital Aarau AG, Tellstrasse 25, 5001 Aarau, Switzerland; 4 Department of Health Sciences and Medicine, University of Lucerne, Lucerne, Switzerland; 5 Department of Pediatrics, McMaster Children’s Hospital and University, Hamilton, Canada; 6 Division of Pediatric Heamtology and Oncology, Paediatric Palliative Care, Children’s Hospital, Inselspital, Universitätsspital Bern, Freiburgstrasse 10, CH-3010 Bern, Switzerland; 7 Paediatric Palliative Care, Children’s Hospital Basel, Spitalstrasse 33, 4056 Basel, Switzerland; 8 Paediatric Palliative Care, Children’s Hospital, Inselspital, Universitätsspital Bern, Freiburgstrasse 10, CH-3010 Bern, Switzerland; 9 Department of Epidemiology and Public Health, Swiss Tropical and Public Health Institute, Kreuzstrasse 2, 4123 Allschwil, Switzerland; 10 University of Basel, Basel, Switzerland; 11 Clinical Trial Unit, Department of Clinical Research, University of Basel, Basel, Switzerland; 12 Department of Biostatistics, Epidemiology, Biostatistics and Prevention Institute, University of Zurich, Hirschengraben 84, 8001 Zurich, Switzerland

**Correction: BMC Palliat Care 21, 188 (2022)**

**https://doi.org/10.1186/s12904-022-01089-x**

Following publication of the original article [1], the author reported that there is an error in the data on page 8.

**The data** “… to ensure a total of **98** evaluable pairs of care giving parents, …” should read“ “… to ensure a total of **88** evaluable pairs of care giving parents, …”“

The original article has been updated.
